# Effectiveness and safety research of Qingfei Paidu (QFPD) in treatment of COVID-19: an up-to-date systematic review and meta-analysis

**DOI:** 10.1186/s13020-022-00675-8

**Published:** 2022-10-28

**Authors:** Xinxin Wang, Tao Ma, Wei Zhang, Qiang Chu

**Affiliations:** 1 Jinling Institute of Technology, Nanjing, 211100 China; 2 Department of Traditional Chinese Medicine, Gusheng Tang Stone Road Clinic, Suzhou, 215031 China; 3 Xinyi People’s Hospital, Xuzhou, 221400 China; 4 Kowloon Hospital, Suzhou, 215000 China

**Keywords:** COVID-19, Effective treatment options for COVID-19, Traditional Chinese medicine(TCM), Qingfei Paidu, Randomized controlled trial, Systematic review, Meta-analysis

## Abstract

**Background:**

Since the outbreak of COVID-19 in 2019, the global economy, culture, politics, and people's lives and health have been severely damaged and threatened. Although western modern medical treatment has made great efforts, the treatment of COVID-19 has not achieved ideal clinical efficacy with severe sequelae. Qingfei Paidu (QFPD), an important herbal prescription for COVID-19 treatment, has shown remarkable therapeutic effects in China's fight against the epidemic.

**Materials and methods:**

We searched seven databases up to 7 September 2022, including PubMed, Chinese National Knowledge Infrastructure (CNKI), Wanfang Database, Cochrane Central Register of Controlled Trials (CENTRAL), EMBASE, World Scientific and SpringerLink. We used the Cochrane Risk of Bias tool to assess the quality of randomized controlled trials. All analysis results were conducted by RevMan 5.4.1 to carry out a meta-analysis.

**Results:**

Fifteen studies with 10390 patients were included. QFPD could not only significantly improve the cure rate and lung CT of COVID-19, reduce the number of patients turning to critical condition and death, shorten the time for nucleic acid conversion and the length of hospital stay, but change laboratory indexes and relieve body symptoms quickly without adverse effects.

**Conclusions:**

Compared with patients only treated by conventional western treatment (CWM), QFPD combined with CWM could be more effective for patients. It is worth spreading to other countries in the global battle against COVID-19.

## Introduction

As of 7 July 2022, over 500 million confirmed cases and over 6 million deaths have been reported globally [1]. According to the Tianmu Global COVID-19 Ranking, China ranks first in the fight against COVID-19, which depends on the correct anti-epidemic policies of the Chinese government, the active cooperation of the Chinese people, and especially the comprehensive intervention of traditional Chinese medicine.

With a history of more than 2000 years, TCM has accumulated rich experience in fighting epidemics, helping Chinese people solve 321 plagues in history, including SARS in 2003 and H1N1 flu in 2009. Likewise, TCM played a great role in fighting COVID-19. For example, X.M Hu summarized the clinical syndromes of COVID-19, including “Cold dampness” and “Yidu”, and retrospectively analyzed the exact clinical efficacy of QFPD combined with western medicine from multiple perspectives [2].

On 31 March 2022, WHO explicitly affirmed the importance of traditional Chinese medicine (TCM) in treating COVID-19, and encouraged WHO member states to learn from the Chinese model of integrated traditional and western medicine. Therefore, we should pay greater attention to the TCM as a gift from China to the world.

QFPD is an important component of traditional Chinese medicine treatment of COVID-19. Although the prescription has been widely used in China and overseas, the majority of the research were case series [3–7] and researchers did not have very much discussion on the mechanism of action of the prescription [8–10], especially the systematic review and meta-analysis of QFPD based on randomized or non-controlled trials [11]. At the root, many clinical randomized controlled trials were conducted after the article was published, resulting in insufficient sample sizes to support systematic review and meta-analysis in the early stages of the COVID-19 outbreak. In addition, the outstanding TCM contribution to COVID-19 has not been recognized by World Health Organization before, so researchers rarely conducted systematic review and meta-analysis of QFPD. These two limitations result in a relative lack of literature on systematic review and meta-analysis of QFPD. Therefore, in order to fill the gap in the literature of systematic evaluation and meta-analysis, and to support the treatment recommendations of WHO, the systematic review and meta-analysis was conducted by evaluating the effectiveness and safety of QFPD in the treatment of COVID-19. The research is of great significance, especially at a time when the global epidemic is becoming worse.

We are very willing to share the treatment method of COVID-19 without reservation, namely QFPD, in the hope that this study can not only give the world a more comprehensive and profound understanding of the effective treatment of traditional Chinese medicine in COVID-19 but also help people in plague-stricken countries and regions recover from serious illness, praying for them to get rid of the disease as soon as possible.

## Methods

The study was conducted and reported in accordance with the guidelines for the Preferred Reporting Items for Systematic Reviews and Meta-Analyses (PRISMA) [12] and Cochrane Handbook for Systematic Reviews [13]. We prospectively registered this study on the International Prospective Register of Systematic Reviews (PROSPERO: CRD42022323735).

### Eligibility criteria

#### Types of studies

All RCTs which have evaluated the efficacy and safety of QFPD for COVID-19 were included in this study.

#### Type of participants

All patients confirmed by relevant diagnostic criteria (COVID-19 Diagnosis and Treatment Protocol Trial Version 9) could be enrolled in this review. In order to ensure that studies could be included as many as possible, we removed gender, age, and national restrictions.

#### Types of interventions

The dosage forms of QFPD mainly include decoction and granule [14]. Patients in the experimental group received treatment of QFPD granule or QFPD combined with conventional western medicine (CMW), while patients in the control group received western medicine treatment. CMW included nutritional support, respiratory support, antiviral treatment, antibacterial drugs, antibiotics, corticosteroids, α-IFN inhalation, symptomatic treatment, and other routine treatments.

In the experimental group and control group, the name of conventional western medicine treatment and the dosage used must be the same. The dosage form, type, quantity and course of QFPD were not restricted. The observation time should be at least 2 weeks.

#### Types of outcome measures

The primary outcome measure was defined as clinical cure rate, lung CT, ranging from mild to critical cases, death, length of hospital stay, time for nucleic acid conversion, and adverse effects; The secondary outcome measure was clinical symptoms, including cough, fever, and fatigue; The third type was laboratory indicators, including White blood cell (WBC), Procalcitonin (PCT), and C-reactive protein (CRP).

### Search strategies

We searched seven databases up to 7 September 2022, including PubMed, Chinese National Knowledge Infrastructure (CNKI), Wanfang Database, Cochrane Central Register of Controlled Trials (CENTRAL), EMBASE, World Scientific, and SpringerLink, which constituted the evidence basis for subsequent data analysis. In order to obtain comprehensive relevant document literature, the retrieval process mainly focused on the titles of medical topics, regardless of the country, region, and language limitations of the article. The following keywords were used to search through various databases: (‘qingfei paidu’ OR ‘Qingfei Paidu’ OR ‘Qing-Fei-Pai-Du’ OR ‘qing fei pai du’) AND(‘COVID-19’ OR ‘corona virus disease 2019’OR ‘2019 novel coronavirus’ OR ‘SARS-Cov-2’ OR ‘novel coronavirus pneumonia’) AND (‘clinical trial’ OR ‘clinical study’ OR ‘randomized controlled trial’ OR ‘RCT’). In order to avoid omission, we also searched the references of the found articles, which were completed by two researchers independently (Wang XX and Ma T).

### Filtering criteria

We designed the inclusion criteria as follows:

- Random controlled trials involving COVID-19 patients treated with QFPD.
- Patients treated with QFPD or QFPD combined with western medicine.
- The article had the scientific data which can judge the efficacy and safety of QFPD.

Excluded criteria were set as follows:

- The experimental group was involved in a variety of Chinese medicine, so it was impossible to determine the exact effect of QFPD alone.
- experimental studies, case reports, reviews, theoretical discussions, commentaries, abstracts, and editorials.
- Studies judged to be unreliable for different reasons

### Study selection

According to the inclusion and exclusion criteria of literature, two researchers (Wang XX and Ma T) imported literatures meeting the requirements into Endnote software. First of all, the two researchers removed articles that did not meet the inclusion criteria by independently scanning the titles, abstracts, and keywords of the articles. Secondly, after downloading the remaining literature, they carefully read the full text to further determine whether it should be included in the study. Finally, the results of the first 2 rounds of screening were cross-checked by 2 researchers. If there is any disagreement, they can discuss it with a third reviewer (Zhang W) to achieve consensus.

### Data extraction and management

To have a more comprehensive understanding of the basic characteristics of the included studies, we prepared an excel form to collect and input relevant information. If important methodological information is lacking or the specific content of the paper is disputed, the researcher should contact the author of the paper to find out. Two researchers (Wang XX and Zhang W) read the literature independently, extracted data, and filled in tables. The basic data extracted mainly included the name of the first author, the publication date of the paper, sample size, number of men and women in the experimental group and control group, age, intervention treatment, treatment duration, and main outcomes. If there is a disagreement in data extraction, a third party (Chu Q) will participate in the discussion and reach a consensus.

### Assessment of risk of bias

The quality assessment of included studies adopted the risk of bias (ROB) assessment tool provided by the Cochrane Handbook. Two researchers (Wang XX and Chu Q) assessed the methodological quality of trials independently. The following seven aspects were evaluated, such as random sequence generation, allocation concealment, blinding of participants and personnel, blinding of outcome assessment, incomplete outcome data, selective reporting, and other bias. According to the above criteria, the evaluation results were ranked as low risk, unclear risk or high risk. Discrepancies were resolved by consulting a third party (Ma Tao) to arrive at a consensus conclusion during the evaluation process.

### Data analysis

In order to conduct random-effects model meta-analysis, we used Review Manager software (RevMan 5.4.1, 2020) as the analytical tool. In the process, for dichotomous data, we calculated the risk ratio (RR) with corresponding 95% confidence interval (95% CI). For continuous data, we used the mean difference (MD) with 95% to represent it. In the case of missing data, we performed estimates according to the Cochrane Handbook for Systematic Reviews of Interventions. Heterogeneity between trials was evaluated with I^2^ statistic. Based on I^2^ values of < 25%, 25–50% and > 50%, heterogeneity was evaluated as low, medium, and high levels, respectively. The analytical model was divided into 2 types, namely, Fixed and Random Effect model. Fixed Effect model was selected when P > 0.1 and I^2^ < 50%. Random Effect model was utilized while P ≤ 0.1 and I^2^ ≥ 50%. A forest plot was used to estimate the effect of an intervention, while a funnel plot was used to evaluate whether there is bias in the meta-analysis. P value < 0.05 is regarded statistically significant.

### Sensitivity analysis

For indicators with high heterogeneity in analysis results, we used Stata 17.0 to conduct sensitivity analysis for them. By removing one study at a time, we analyzed the other studies to estimate whether the results might have been significantly affected by a single study.

### Evidence quality evaluation (GRADE)

Grades of Recommendations Assessment, Development and Evaluation (GRADE) was used to assess the quality of evidence for WM intervention alone versus combined QFPD-WM intervention. We imported the data from Revman5.4 into GRADEpro guide development tool to build an evidence table. The initial evidence strength was set as high because all studies included in this meta-analysis were RCTs.

The GRADE approach assesses the quality of the level of evidence from 5 considerations (study limitations, consistency of effect, imprecision, indirectness and publication bias). Cochrane Handbook recommends choosing up to 7 main outcomes that are essential for decision-making. Consequently, we chose 7 important outcomes, including clinical effective rate, lung CT, ranging from mild to critical, time for nucleic acid conversion, adverse effect, fever, and CRP according to the requirement of Cochrane.

## Results

### Study selection

**Fig. 1:**
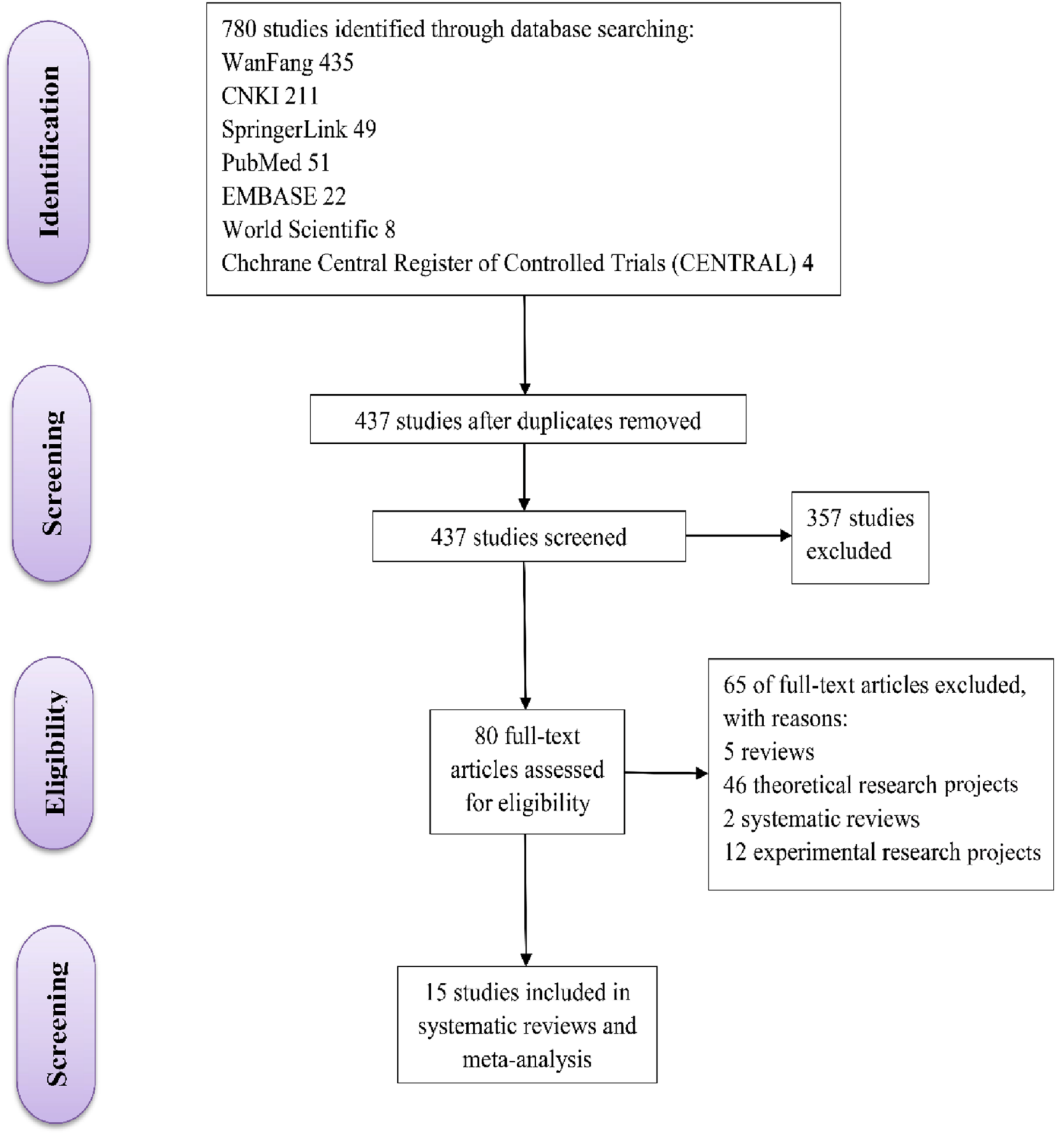
Flow diagram of study selection process

As shown in Fig. 1, a total of 780 relevant literatures were found in the seven databases mentioned above in the process of literature retrieval and screening. After removing duplicate articles, 437 papers remained. Then, 357 publications were excluded as most of them did not meet the inclusion criteria. After browsing and checking, the remaining 80 articles were screened again according to the research direction.80 articles were further excluded: 5 reviews; 46 theoretical research projects; 2 systematic reviews; 12 experimental research projects. Finally, 15 studies were assessed to be eligible and included in our review.

### Study characteristics

Table 1 showed the basic characteristics of the finally included research objects. Three of the articles were in English and the rest were in Chinese. There were a total of 10390 COVID-19 patients (male: 47.29%) in the 15 studies included. All clinical controlled trials have been conducted in China since 2019. The disease stages of the patients included in the study were mild, moderate, severe, and critical. No trial utilized placebo of QFPD.

**Table 1:** Basic characteristics of included studies

No	References			Sample size(T/C)	T/C (M/F)	Age (T/C)	Treatment		Treatment duration (T/C)	Main outcomes
							Intervention	Control		
1	Yu, et al. [21]			102(64/38)	T(45/19) C(19/19)	46.5(20–68) 51.5(21–69)	QFPD	CWM treatment	10/10.5	A1, A4
2	Geng, et al. [15]			75(47/28)	T(22/25) C(13/15)	40.26 ± 12.33/39.47 ± 13.46	QFPD + CWM treatment	CWM treatment	9/9	A2, A3, A4, B1, B2, B3
3	Wang, et al. [25]			72(42/30)	T(20/22) C(15/15)	40.42 ± 12.59/41.79 ± 13.42	QFPD + CWM treatment	CWM treatment	16.20 ± 5.57/22.98 ± 5.84	A2, A3, A6, C1
4	Zhang, Pan [22]			24(12/12)	T(6/6) C(7/5)	61.42 ± 13.24/62.25 ± 14.69	QFPD + CWM treatment	CWM treatment	7/7	A1,A7,C1,C2, C3
5	Zeng, Ma, Wang et al. [24]			229(104/125)	T(56/48) C(68/57)	46.65 ± 6.21/46.21 ± 5.62	QFPD + CWM treatment	CWM treatment	24.63 ± 2.31/29.35 ± 2.47	A2, A3, A7, C1
6	Wang, et al. [14]			140(70/70)	T(35/35) C(36/34)	48.00 ± 13.2/49.4 ± 13.3	QFPD + CWM treatment	CWM treatment	7.03 ± 1.21/10.13 ± 2.36	A1, A3, A4, A6, A7, C1, C2
7	Yu, et al. [26]			89(43/46)	T(21/22) C(18/28)	64.23 ± 2.51/60.50 ± 2.08	QFPD + CWM treatment	CWM treatment	26.86 ± 1.76/33.46 ± 1.59	A2, A3, A6, C2
8		Li, Zhang [18]	12(6/6)		T(4/2) C(3/3)	52.00 ± 6.56/50.00 ± 10.00	QFPD + CWM treatment	CWM treatment	11.45 ± 4.58/19.40 ± 7.02	A1, A3, A6, A7, C1
9		Li, et al. [17]	60(30/30)		T(15/15) C(13/17)	53.600 ± 0.259/50.433 ± 0.338	QFPD + CWM treatment	CWM treatment	13.633 ± 0.398/16.433 ± 0.295	A1, A3, A4, A6, A7, B1, B2,
10		Sun [19]	62(31/31)		T(18/13) C(14/17)	47.12 ± 6.14/46.57 ± 6.08	QFPD + CWM treatment	CWM treatment	12/12	A1, C2, C3
11		Xu, et al. [10]	37(12/25)		T(6/6) C(13/12)	NR	QFPD + CWM treatment	CWM treatment	12.2/16.8	A2, A3
12		Yang, Sun, Liu. [29]	40(20/20)		T(13/7) C(12/8)	50.16 ± 5.83/49.57 ± 5.46	QFPD + CWM treatment	CWM treatment	5.64 ± 2.03/5.51 ± 2.16	B1, B2, B3, C1
13		Zhang, et al. [27]	8939(2568/6371)		T(1198/1370) C(2970/3410)	NR	QFPD + CWM treatment	CWM treatment	NR	A5, A6
14		Xin, et al. [20]	63(37/26)		T(17/20) C(12/14)	40.1/50.7	QFPD + CWM treatment	CWM treatment	19/17	A1, A3, A5, A7, C3
15		Liu, et al. [23]	446(223/223)		T(111/112) C(113/110)	60 (51–66) /62(50–70)	QFPD + CWM treatment	CWM treatment	NR	A1, A5

*T* trial; *C* control; *M* male; *F* female; *NR* Not reported; *CWM* conventional western medicine; *A*1 clinical cure rate; *A*2 time for nucleic acid conversion; *A*3 length of hospital stay; *A*4 ranging from mild to critical cases; *A*5 death; *A*6: adverse effects; *A*7: lung CT; *B*1: cough; *B*2: fever; *B*3: fatigue; *C*1 WBC; *C*2 CRP; *C*3 PCT

### Assessment of risk of bias

**Fig. 2:**
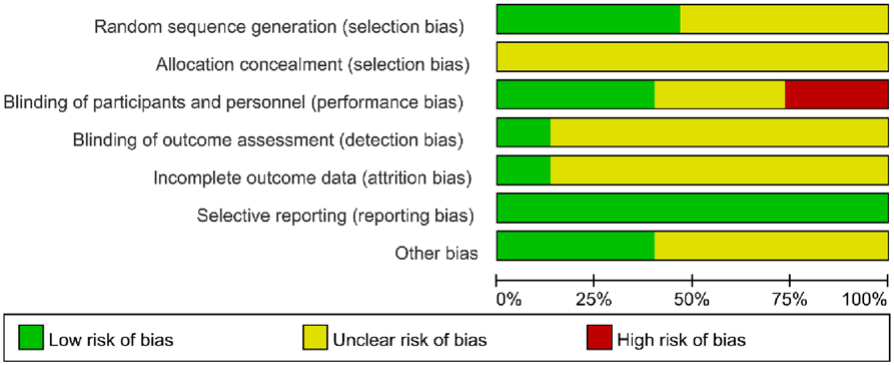
Risk of bias graph

As shown in Table 2 and Fig. 2, the quality assessment of the included studies adopted the risk of bias (ROB) assessment tool provided by the Cochrane Handbook. 7 trials (7/15, 46.7%) [14, 18, 19, 23–25, 27] reported random sequence generation. Allocation concealment was unclear for it was not described in this review. 4 trials reported no application of blinding. 6 trials reported blinding of participants and personnel, and 2 trials [19, 21] reported blinding of outcome assessment. We evaluated each of the included studies as a whole according to these seven criteria: 5 trials were medium risk and the other 10 were at low risk.

**Table 2:** The risk of bias of included trials

References	A	B	C	D	E	F	G	H
Geng LM et al. [15]	?	?	?	?	+	+	+	L
Li KY et al. [17]	?	?	+	?	?	+	?	L
Li YD et al. [18]	+	?	?	?	?	+	?	L
Sun TF et al. [19]	+	?	+	+	?	+	+	M
Wang QL et al. [25]	+	?	−	?	+	+	?	M
Wang Y et al. [14]	+	?	−	?	?	+	?	M
Xin SY et al. [20]	?	?	−	?	?	+	?	M
Xu TL et al. [28]	?	?	+	?	?	+	?	L
Yang M et al. [29]	?	?	−	?	?	+	+	M
Yu HY et al. [21]	?	?	?	+	?	+	?	L
Yu XY et al. [26]	?	?	+	?	?	+	+	L
Zeng XH et al. [24]	+	?	?	?	?	+	?	L
Zhang LH et al. [27]	+	?	+	?	?	+	+	L
Zhang P et al. [22]	?	?	?	?	?	+	?	L
Zhen L et al. [23]	+	?	+	?	?	+	+	L

*A* Random sequence generation; *B* Allocation concealment; *C* Blinding of participants and personnel; *D* Blinding of outcome assessment; *E* Incomplete outcome data; *F* Selective reporting; *G* Other bias; + Low risk;—High risk; ? Unclear; *L* Low; *M*Medium

### Description of QFPD

QFPD consists of 21 traditional Chinese herbs as shown in Table 3. Ephedra, containing ephedrine and pseudoephedrine, has the effect of epinephrine, and it excites sympathetic nerves, promotes sweating, and removes evil to go out. Glycyrrhiza uralensis Fisch invigorates the stomach and reconciles various drugs. In addition, glycyrrhizic acid has the effect of corticosteroids and plays a strong anti-inflammatory effect. Pericarpium citri Reticulatae, agastache rugosus, and ginger contain volatile oil, sweating while promoting intestinal peristalsis. Its luteolin (Fig. 9b) can reduce the production of pro-inflammatory cytokines and inflammatory mediators, and regulate immunity [30]. Asarum contains kaempferol, anti-inflammatory, anti-cancer, anti-oxidation, antiviral, and anti-bacterial. It can enhance the body's immunity and has other pharmacological effects. Kaempferol showed concentration-dependent inhibition in non-cytotoxic concentrations [31]. Yam promotes digestion and nourishes the body, and a large amount of starch helps dissolve the effective components of raw gypsum.

**Table 3:** Components of Qingfei Paidu decoction

Chinese name	Latin name	Dose (g)
Ma Huang	Ephedra	9
Zhi Gan Cao	Glycyrrhiza uralensis Fisch	6
Xing Ren	Amygdalus communis	9
Bai Zhu	Atractylodes macrocephala Koidz	9
Chai Hu	Radix bupleuri	16
Huang Qin	Scutellaria baicalensis	6
Jiang Ban Xia	Pinellia ternata	9
Zi Wan	Asteris radix et rhizoma	9
Kuan Dong Hua	Farfarae flos	9
She Gan	Belamcanda chinensis	9
Xi Xin	Asarum	6
Shan Yao	Dioscorea polystachya	12
Zhi Shi	Citrus aurantium	6
Huo Xiang	Agastache rugosus	9
Sheng Jiang	Zingiber officinale Rosc	15
Fu Ling	Wolfiporia cocos	15
Chen Pi	Pericarpium citri Reticulatae	6
Sheng Shi Gao	Raw Gypsum	15–30^a^
Gui Zhi	Cinnamomum cassia Presl	9
Ze Xie	Alismatis	9
Zhu Ling	Polyporus umbellatus	9

^a^30 g is for patients with fever

In summary, QFPD can repair lung injury, enhance the immunity of the body, and play a role in regulating multiple targets and signaling pathways. Pharmacological studies have shown that QFPD inhibits the mRNA translation of the SARS-COV-2 virus by acting on multiple ribosomal proteins [32]. The dosage forms of QFPD include decoction (14/15, 93.3%) and granule (1/15, 6.7%).

### Overall outcomes assessment

#### Clinical cure rate

Thecriteria for clinical cure rate must meet the four conditions:

- The body temperature has returned to normal for more than 3 days;
- Respiratory symptoms improved significantly;
- Lung imaging showed significant absorption improvement of acute inflammation;
- Two consecutive negative nucleic acid tests of respiratory tract specimens (The sampling interval must be at least one day) (COVID-19 Diagnosis and Treatment Protocol Trial Version 9) [33].

**Fig. 3:**
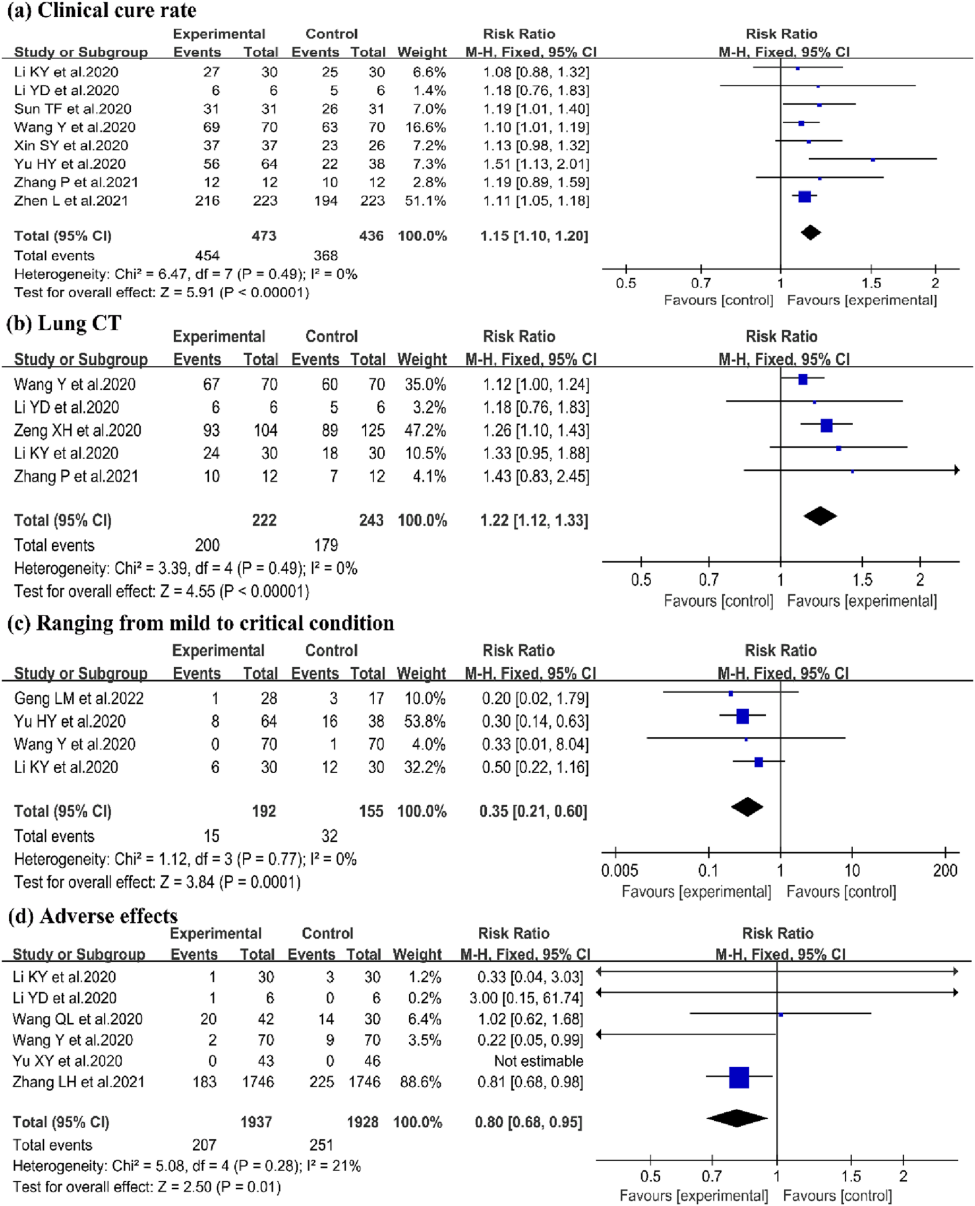
Forest plot of (**a**) Clinical cure rate, (**b**) Lung CT, (**c**) Ranging from mild to critical condition, (**d**) Length of hospital stay

The clinical cure rate of QFPD was reported in 8 studies [14, 17–23]. There were 473 patients in the experimental group and 436 in the control group. Meta-analysis showed that QFPD had a significant improvement on clinical cure rate, compared to CWM (8 trials, n = 909; RR = 1.15; 95%Cl 1.10–1.20; I^2^ = 0%, P < 0.00001; Fig. 3a).

#### Lung CT

Five trials assessed the efficacy of QFPD on lung CT [11, 14, 17, 18, 22]. There were 222 patients in experimental group and 243 in control group. QFPD exhibited a significant improvement on lung CT (5 trials, n = 465; RR = 1.22; 95% Cl 1.12–1.33; I^2^ = 0%, P < 0.00001; Fig. 3b).

#### Ranging from mild to critical condition

In order to evaluate the effects of QFPD on ranging from mild to critical condition, four trials were enrolled in this study [14, 15, 17, 21]. QFPD had obvious important effect on ranging from mild to critical condition (4 trials, n = 347; RR = 0.35; 95%Cl 0.21 to 0.60; I^2^ = 0%, P = 0.0001; Fig. 3c).

#### Adverse effects

The adverse effects were reported in 6 trials [14, 17, 18, 25–27]. Meta-analysis showed that QFPD had a significant improvement on reducing adverse effects (6 trials, n = 3865; RR = 0.8; 95%Cl 0.68–0.95; I^2^ = 21%, P = 0.01; Fig. 3d).

**Fig. 4:**
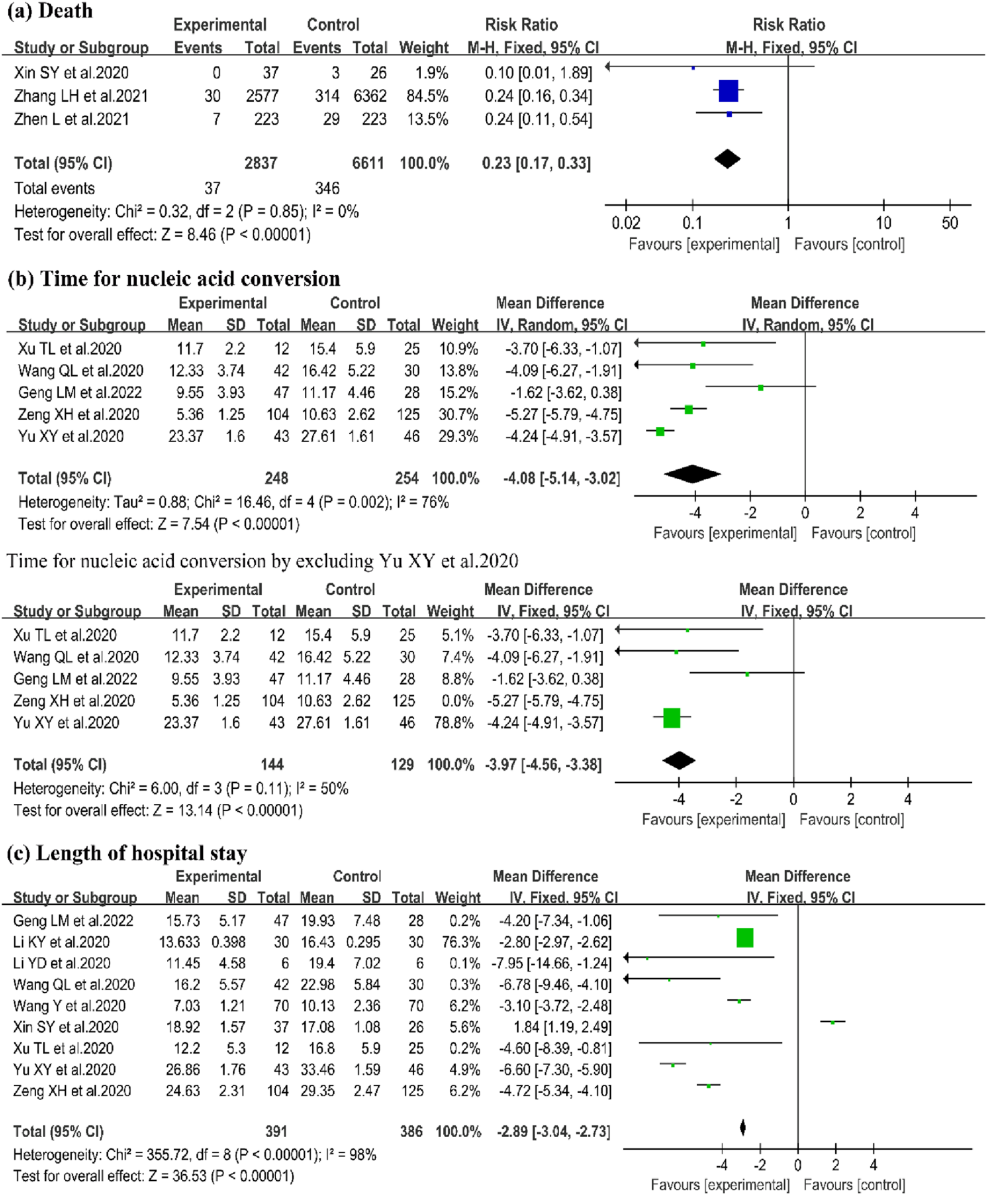
Forest plot of (**a**) Adverse effects, (**b**) Death, (**c**) Time for nucleic acid conversion

#### Death

Three trials evaluated the effects of QFPD on death [20, 23, 27]. Compared to CWM, a significant reduction in death was observed by QFPD (3 trials, n = 9448; RR = 0.23; 95%Cl 0.17–0.33; I^2^ = 0%, P < 0.00001, Fig. 4a).

#### Time for nucleic acid conversion

The effect of QFPD on time for nucleic acid conversion was reported in 5 trials [15, 24–26, 28].QFPD shortened the time for nucleic acid conversion obviously (5 trials, n = 502; WMD = − 4.08; 95%Cl − 5.14 to − 3.02; I^2^ = 76%,P < 0.00001, Fig. 4b).

#### Length of hospital stay

9 studies reported length of hospital stay [14, 15, 17, 18, 20, 21, 24–26]. There were 391 patients in experimental group and 386 in control group. Meta-analysis showed a significant improvement on length of hospital stay by QFPD (9 trials, n = 777; WMD = − 2.89; 95% Cl − 3.04 to − 2.73; I^2^ = 98%,P < 0.00001; Fig. 4c).

### Clinical symptoms assessment

#### Cough

**Fig. 5:**
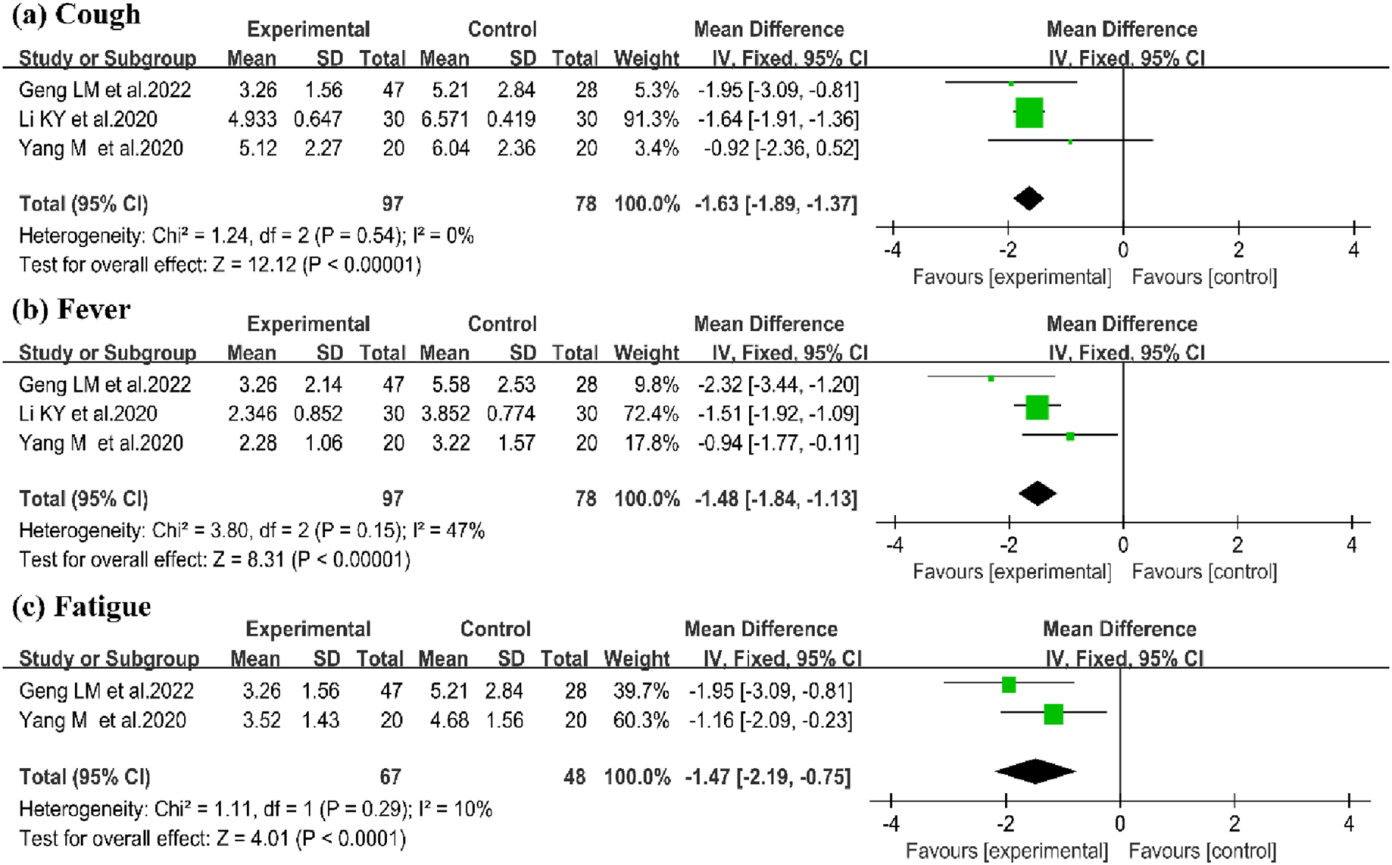
Forest plot of impact of QFPD on (**a**) Cough (**b**) Fever (**c**)Fatigue

In the field of disappearing time of cough, 3 studies were enrolled in the review [15, 17, 29]. A significant improvement on disappearing time of cough between QFPD and CWM was identified in this study (3 trials, n = 175; WMD:-1.63; 95% CI − 1.89 to − 1.37; I^2^ = 0%, P < 0.00001; Fig. 5a).

#### Fever

Three studies reported the symptom of fever [15, 17, 29]. In the field of fever reduction time, the results suggested no significant difference on the time of fever reduction between QFPD and CWM (3 trials, n = 175; WMD: − 1.48; 95% CI − 1.84 to − 1.13; I^2^ = 47%, P < 0.00001;Fig. 5b).

#### Fatigue

The effect of QFPD on fatigue was evaluated in 2 studies [15, 29]. There were 67 patients in experimental group and 48 in CWM group. Improvement on disappearing time of fatigue was identified in QFPD group compared to CWM group (2 trials, n = 115; WMD: − 1.47; 95% CI − 2.19 to − 0.75; I^2^ = 10%, P < 0.0001; Fig. 5c).

### Laboratory indicators

#### WBC

**Fig.6:**
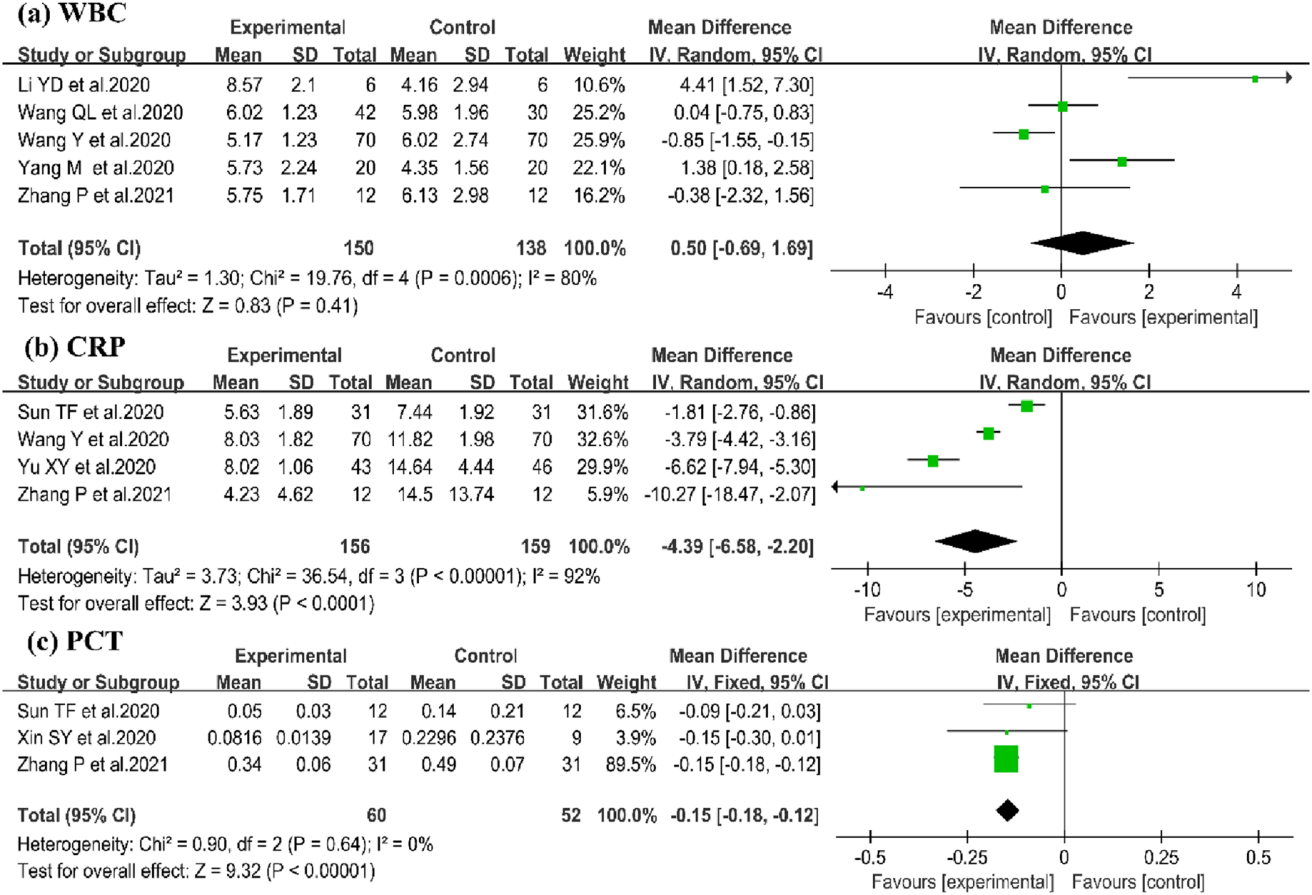
Forest plot of impact of QFPD on (**a**) WBC, (**b**) CRP, (**c**) PCT

For the number of WBC, 5 trials [14, 18, 22, 25, 29] involving 288 patients were enrolled. No significant difference on WBC was identified, compared to CWM (5 trials, n = 288; WMD: 0.50; 95%Cl − 0.69 to 1.69; I^2^ = 80%, P = 0.41; Fig. 6a).

#### CRP

Four trials evaluated the efficacy of QFPD on the level of CRP [14, 19, 22, 26]. Meta-analysis showed a significant improvement between QFPD and CWM on the number of CRP in patients with COVID-19 (4 trials, n = 315; WMD:-4.39; 95%CI − 6.58 to − 2.20; I^2^ = 92%, P < 0.0001; Fig. 6b).

#### PCT

To evaluate the effects of QFPD on the PCT number, 3 trials were enrolled in this study [19, 20, 22]. There were 60 patients in the experimental group and 52 in the control group. QFPD had obvious important effect on PCT (3 trials, n = 112; WMD =− 0.15; 95% Cl − 0.81 to − 0.12; I^2^ = 0%,P < 0.00001; Fig. 6c).

#### Results of sensitivity analysis

**Fig. 7:**
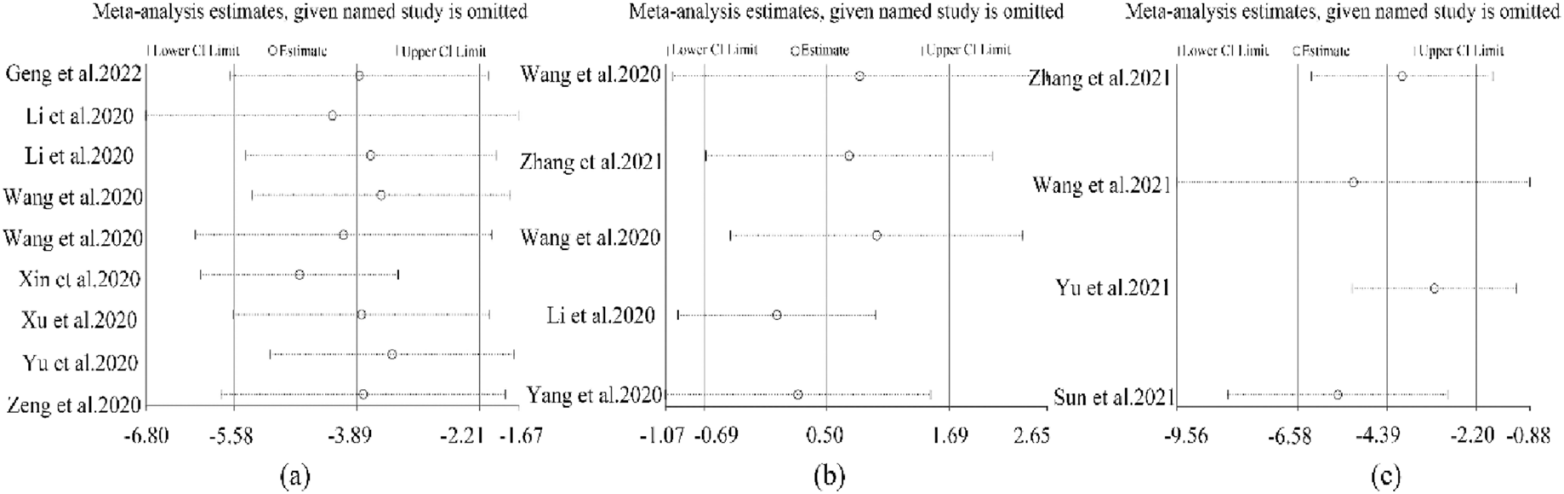
Sensitivity analysis of (**a**) Length of hospital stay, (**b**) WBC, (**c**) CRP

The sensitivity analysis of length of hospital stay, WBC and CRP was shown in Fig. 7, and the results showed that omitting individual studies exhibited no significant effects on the pooled results compared with the results of the original forest map, indicating that the study results were stable.

#### Publication bias

**Fig. 8:**
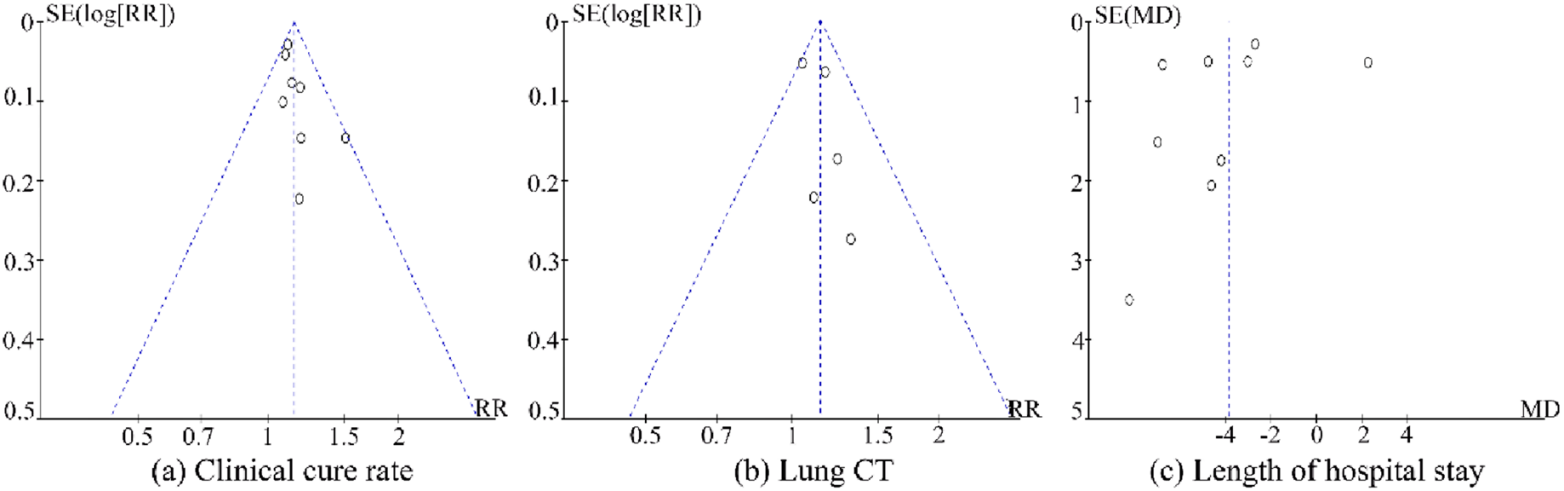
Funnel plot of (**a**) Clinical cure rate, (**b**) Lung CT, (**c**) Length of hospital stay

Publication bias was detected by plotting the funnel plots of included trials. The asymmetry showed a mild publication bias. (Fig. 8).

### GRADE assessment

According to the GRADE assessment for the efficacy and safety of QFPD, different quality levels of evidence were reported in Table 4. There were moderate evidences in clinical effective rate, ranging from mild to critical, adverse effects and CRP. The criteria of “risk of bias” ranked “serious” for all outcome measures, leading the evidence strengths to moderate. Even worse, significant publication bias was also detected in terms of lung CT, time of nucleic acid conversion and fever, which decreased the evidence strength to low. However, the inconsistency, indirectness and imprecision were not serious. The overall evidence strength was assessed as moderate.

**Table 4:** GRADE assessment for combined QFPD-WM vs WM treatment

Outcome	Certainty assessment					Summary findings				Evidence strength
	Risk of bias	Inconsistency	Indirectness	Imprecision	Publication bias	No. of patients		Effect		
						[Intervention]	[Comparison]	Relative (95% CI)	Absolute (95% CI)	
Clinical effective rate	Serious	Not serious	Not serious	Not serious	None	454/473 (96.0%)	368/436 (84.4%)	RR 1.15 (1.10 to 1.20)	127 more per 1000 (from 84 to 169 more)	⨁⨁⨁◯ Moderate
Lung CT	Serious	Not serious	Not serious	Not serious	Publication bias strongly suspected	200/222 (90.1%)	179/243 (73.7%)	RR 1.22 (1.12 to 1.33)	162 more per 1000 (from 88 to 243 more)	⨁⨁◯◯ Low
Ranging from mild to critical	Serious	Not serious	Not serious	Not serious	None	15/192 (7.8%)	32/155 (20.6%)	RR 0.35 (0.21 to 0.60)	134 fewer per 1000 (from 163 to 83 fewer)	⨁⨁⨁◯ Moderate
Time for nucleic acid conversion	Serious	Not serious	Not serious	Not serious	Publication bias strongly suspected	248	254	–	MD 4.08 lower (5.14 lower to 3.02 lower)	⨁⨁◯◯ Low
Adverse effect	Serious	Not serious	Not serious	Not serious	None	207/1937 (10.7%)	251/1928 (13.0%)	RR 0.80 (0.68 to 0.95)	26 fewer per 1000 (from 42 to 7 fewer)	⨁⨁⨁◯ Moderate
Fever	Serious	Not serious	Not serious	Not serious	Publication bias strongly suspected	97	78	–	MD 1.48 lower (1.84 lower to 1.13 lower)	⨁⨁◯◯ Low
CRP	Serious	Not serious	Not serious	Not serious	None	156	159	–	MD 4.39 lower (6.58 lower to 2.2 lower)	⨁⨁⨁◯ Moderate

## Discussion

### Summary of the main results

WHO Director-General Tedros Adhanom Ghebreyesus expressed that he was willing to jointly carry out evidence-based evaluation of TCM in the treatment of COVID-19 with China. As far as we know, our study is the first systematic review and meta-analysis of all published RCTs to evaluate the efficacy and safety of QFPD in the treatment of COVID-19, which greatly fills the gap in the field of evidence-based medicine for the treatment.

The study came from 15 experiments involving 10390 patients. Although QFPD plays an important role in the treatment of COVID-19, we should rationally view the quality of evidence for QFPD. Firstly, to draw a better conclusion, the articles collected in this systematic review were all RCTs to ensure the strength of the evidence. However, the results were not ideal, so we did some remedial measures to explore the details of heterogeneity with the principle of scientific research. For the high heterogeneity of some indicators, we chose the random effect model and pooled the effect index. By adjusting the weight of the included studies, studies with large samples were given less weight while studies with small samples were given more weight, which can partially reduce the influence of heterogeneity. Furthermore, we introduced the method of sensitivity analysis. After we removed the included studies one by one, the combined effect values were all within the confidence interval range. There were no reversals or apparent fluctuations in the observed results, so the analysis can be regarded as relatively stable. Additionally, it was found that the only existing systematic review of multiple literature included 7 trials. By contrast, our review is more comprehensive and reliable. While further research projects are still needed, these data are valuable and timely, given that there are no special drugs and the high infectious rate.

In addition, in order to make full use of the data and supplement the deficiency, we conducted a scientific and reasonable treatment of the data: for experiments without mean value and standard deviation, the sample capacity, median, maximum and minimum value were used to estimate such as the age of some patients and other data [34, 35].

Then, with the purpose of evaluating the efficacy of QFPD comprehensively, three categories of indicators were included. The first type was overall outcomes assessment, including clinical cure rate, lung CT, ranging from mild to critical cases, death, length of hospital stay, time for nucleic acid conversion, and adverse effects. The secondary outcome measure was clinical symptoms, including cough, fever, and fatigue. The third type was laboratory indicators, including White blood cell (WBC), Procalcitonin (PCT), and C-reactive protein (CRP).

Serum CRP was abnormally high in severe COVID-19 patients and was positively correlated with the severity of COVID-19 patients. Meanwhile, the PCT level in critical patients was significantly higher than that in healthy patients. Compared with the control group, CRP and PCT were significantly reduced after QFPD intervention.

Moreover, regarding the safety of QFPD, a total of 6 studies investigated side effects. The majority of experiments have proved that QFPD has no side effects. Only 2 studies have provided the manifestation and data statistics of side effects, including dyslipidemia and diarrhea, but they were unpersuasive considering that side effects were mild, short-term, and in the minority. Therefore, we have every reason to believe that QPFD is relatively safe in the treatment of COVID-19.

**Fig. 9:**
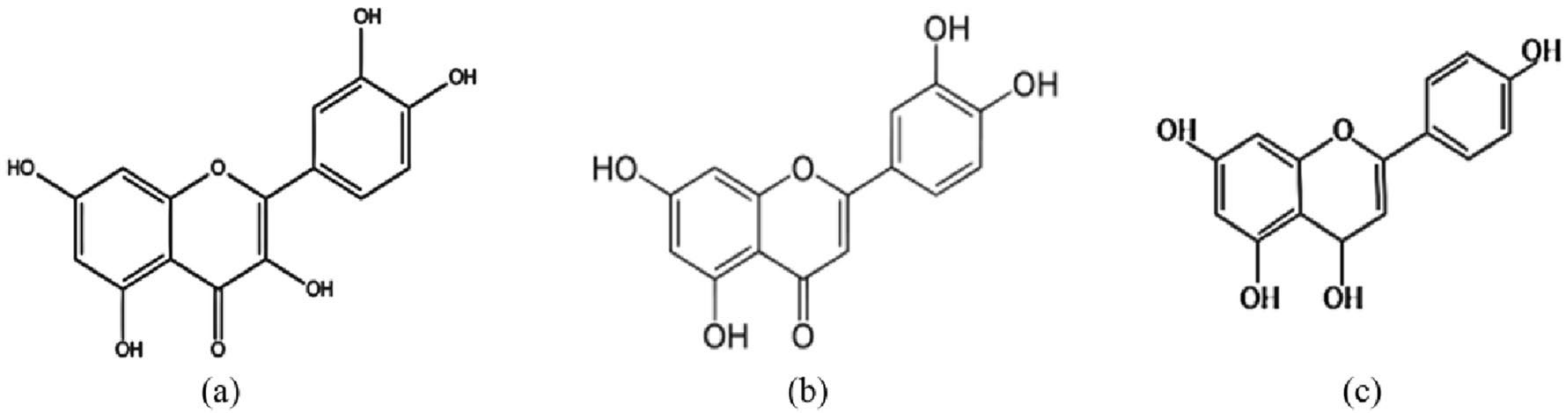
The first 3 main active ingredients of Qingfei Paidu

Last but not least, QFPD was obtained by modifying the combination of four classic prescriptions: Maxing Shigan decoction, Shegan Mahuang decoction, Xiao Chaihu decoction and Wuling powder. In the prescription, Cinnamomum cassia Presl dilates blood vessels, antipyretic and analgesic, and plays a synergistic role with ephedra. Citrus Aurantii has the effects of breaking stagnant qi and removing food retention and reducing phlegm. Naringin (Fig. 9c) in it, as a flavonoid, has antibacterial, anti-inflammatory and antiviral effects, including inhibiting the growth of Staphylococcus aureus, Escherichia coli and Zika virus (ZIKV) [36]. Amygdalus communis, aster, farfarae flos, pinellia ternata, and Scutellaria baicalensis, are anti–inflammation and anti-virus. Polyporus, Alisma and Poria are diuretic and regulate body water metabolism. Ergosterol (Polyporus) and 23-acetate B (Alismatis) could act directly on 3CLpro of novel coronavirus-19, thereby blocking virus proliferation [37]. Radix Bupleuri soothes the liver and enhances the immune-killing function of antigen presenting cells (APC) in the liver. It contains quercetin (Fig. 9a), expectorant and relieving cough, and plays a role in anti-fibrosis [38]. Raw gypsum quenches thirst and promotes fluid production, clears heat and purges fire. The compounds of QFPD bind to six host proteins that interact with the SARS-COV-2 protein, further supporting the antiviral effect of QFPD. It can regulate six biological processes (pattern recognition receptors, cell apoptosis, and clotting, biological oxidation and arachidonic acid metabolism) and four organ systems (nerve, sensory, circulation and digestive system) corresponding to the network of 10 targets [39]. In a word, QFPD is anti-infection and plays an important role in anti-inflammatory effect on immune regulation, multiple organ and systemic protection. These studies provide solid evidence for the treatment of COVID-19 by QFPD.

## Limitations

It is worth noting that this review still has certain limitations as follows. First of all, all the studies are from China, and the samples of randomized controlled studies are still relatively small, which makes it difficult to carry out subgroup analysis, multi-level meta-analysis and other clinical research methods. Secondly, few main outcomes were discussed in some studies, leading to fewer indicators of treatment outcomes presented in the forest map, and some publication bias was difficult to obtain, which should be treated with caution. Additionally, it is common for viral nucleic acid tests to be positive again during the recovery phase of COVID-19, but regrettably few trials conducted long-term follow-up. Objectively, although this meta-analysis shows that QFPD has advantages in treating COVID-19 in many aspects, the quality of included RCTs is not high, or even low in some parts according to the GRADE assessment. Considering the evidence-based medicine of traditional Chinese medicine is still in its infancy, and clinical doctors also lack consciousness, it’s understandable that the overall evidence level is not ideal. Therefore, more high-quality studies are needed to prove the efficacy and safety of TCM and guide clinical practice, as we strongly anticipate.

Despite these limitations, due to the urgency of the COVID-19 pandemic and the necessity for evidence-based medicine information, this meta-analysis must be performed to save more lives.

## Conclusions

In summary, our systematic review and meta-analysis indicated that QFPD could be not only effective in improving clinical cure rate, lung CT, reducing the number of patients with mild to critical condition and death significantly, but also decreasing the time for nucleic acid conversion, shortening the length of hospital stay, reducing the duration of clinical symptoms and improving the laboratory indexes. No obvious adverse effects related to QFPD were identified. QFPD could be used as a more effective treatment option for COVID-19 to spread to other western countries in this battle.
